# Using Rapid-Cycle Change to Improve COVID-19 Vaccination Strategy in Primary Care

**DOI:** 10.3390/ijerph20042902

**Published:** 2023-02-07

**Authors:** Lindsay S. Hunt, Erin E. Sullivan, Jordan Susa, Roger Chaufournier, Claudine Joseph, Russell S. Phillips, Kirsten Meisinger

**Affiliations:** 1 Meadows Mental Health Policy Institute, Dallas, TX 75214, USA; 2 Sawyer Business School, Suffolk University, Boston, MA 02108, USA; 3 Center for Primary Care, Harvard Medical School, Boston, MA 02115, USA; 4 Department of Global Health and Social Medicine, Harvard Medical School, Boston, MA 02115, USA; 5 Diversity by Doing HealthTech, Mountain View, CA 94040, USA; 6 Department of Medicine, Beth Israel Deaconess Medical Center, Boston, MA 02115, USA; 7 Cambridge Health Alliance, Cambridge, MA 02143, USA

**Keywords:** COVID-19, vaccination, primary care, rapid change

## Abstract

During the COVID-19 pandemic, misinformation and distrust exacerbated disparities in vaccination rates by race and ethnicity throughout the United States. Primary care, public health systems, and community health centers have shifted their vaccination outreach strategies toward these disparate, unvaccinated populations. To support primary care, we developed the SAVE Sprint model for implementing rapid-cycle change to improve vaccination rates by overcoming community outreach barriers and workforce limitations. Participants were recruited for the 10-week SAVE Sprint program through partnerships with the National Association of Community Health Centers (NACHC) and the Resilient American Communities (RAC) Initiative. The majority of the participants were from community health centers. Data were evaluated during the program through progress reports and surveys, and interviews conducted three months post-intervention were recorded, coded, and analyzed. The SAVE Sprint model of rapid-cycle change exceeded participants’ expectations and led to improvements in patient education and vaccination among their vulnerable populations. Participants reported building new skills and identifying strategies for targeting specific populations during a public health emergency. However, participants reported that planning for rapid-pace change and trust-building with community partners prior to a health care crisis is preferable and would make navigating an emergency easier.

## 1. Introduction

The U.S. has experienced both successes and failures with vaccination campaigns since the 1940s. Lessons from a successful smallpox vaccine rollout in the late 1940s demonstrated high uptake due to vaccines given by healthcare providers at schools, and the public’s trust in the medical community. The 1955 polio vaccine distribution was also successful in terms of polio cases prevented via vaccination, but issues with unequal vaccine access were apparent; it was less likely that African American children received the polio vaccine [1]. More recently, the 2009 vaccination campaign for H1N1 did not ramp up to full scale once it was discovered that H1N1 was not as deadly as traditional influenza; the anti-vaccination movement impacted public ambivalence to the H1N1 vaccine [1]. This context laid the historical groundwork for the challenges and strategies to vaccinate populations for COVID-19, when skepticism around vaccines and public health systems pervaded at unprecedented levels.

Vaccines significantly decrease COVID-19 mortality, morbidity, and transmission [2]. Developing and distributing a vaccine within a remarkably short timeframe was one of the major logistical challenges of the COVID-19 pandemic [3]. These particular COVID-19-related challenges were in addition to the usual barriers to vaccine uptake: cost, insurance coverage, safety concerns, lack of education, risks associated with the vaccine, and lack of clinicians recommending the vaccine [4]. To overcome some logistical hurdles, community health centers and rural, critical access hospitals across the nation worked to create new workflows and rapidly administer vaccines in their communities, based on the amount of vaccines available to them [5,6]. However, evidence about COVID-19 vaccinations demonstrated clear disparities in COVID-19 vaccination rates, cases, and related deaths by race and ethnicity when adjusted for age [7,8,9]. Several of the vaccine options required high-grade storage and utilization requirements, which made equitable vaccine distribution among health care providers difficult [10]. Additionally, widespread vaccine misinformation associated with vaccine hesitancy undermined the efforts of primary care, public health, and community-based organizations to achieve the desired rates of vaccination across all populations [11,12]. COVID-19 vaccine misinformation spread via social media was most related to safety concerns and conspiracy theories, which Ngai et al. suggested reflects the government and health organization efforts to highlight the safety of the COVID-19 vaccine [12]. 

The initial rush to vaccinate the eligible populations subsided, leaving some traditionally vulnerable groups unvaccinated, such as low-income and rural households [5]. Primary care, community-based organizations, local public health departments, and community health centers have continued efforts to fill the gaps in vaccine promotion and outreach [6]. Numerous strategies and approaches to increasing vaccination rates were promoted online and through educational web-based calls, yet these required the time and energy of health care providers—a resource that was in short supply [13]. At the same time, there was also a remarkable shortage of people coming forward to get vaccinated in many US communities [14]. Health care needed a way to reach community members to remove any remaining barriers to vaccination and establish trusting relationships with communities that had high levels of mistrust of vaccines [15]. The [deidentified for peer review] Sprint to Accelerate Vaccines Equitably (SAVE Sprint) in the summer 2020 was designed as an urgent response to vaccinate vulnerable communities; vulnerable communities were considered those at risk of poor physical, psychological, and/or social health [16]. 

There are many strategies and approaches to increasing vaccination rates [17], but none (to our knowledge) combine expert knowledge, data tracking, a focus on equity, and continuous improvement in an accelerated framework. Our SAVE Sprint model was different from the traditional ‘Breakthrough Series Model’ [18] or Learning Collaborative approach, which is typically six to 15 months [19]. The SAVE Sprint model was originally adapted from the Breakthrough Series Model, but ours was designed to be faster paced and more focused. While a learning collaborative might focus on a complex topic involving several changes (such as improving flow through the ED or mental health integration into primary care), a Sprint is focused on one specific area of improvement. We used the principles of the Lean ‘Rapid Improvement Event’ or ‘Scrum Event’, where a small team of stakeholders focus on making improvements in a specific area over a short intense period [20]. 

## 2. Materials and Methods

### 2.1. Program Design

The aim of the SAVE Sprint was to increase COVID-19 vaccination rates for at-risk populations using a new model for change. The SAVE Sprint program design team included a clinician, a data specialist, two educators with expertise in improvement methodology, and a patient partner. In recognition of the importance of racial and ethnic representation for this work, 66% of the program teams were BIPOC (Black, Indigenous, and People of Color). 

The factors that informed the educational program design included: (1) the reluctance of health care organizations to commit to a program longer than several weeks due to COVID-19-related stress and extreme workloads; (2) the need for a fully-remote program model; (3) the benefits of peer-based learning and support; (4) the recognition that usual approaches to vaccination were not successful; and (5) the urgent unanswered questions related to pandemic-related changes and challenges.

#### 2.1.1. Vaccine Intervention Model

**Figure 1:**
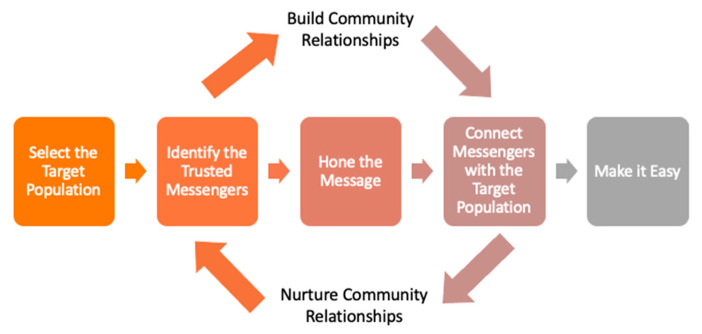
Sprint Vaccine Intervention Model.

The SAVE Sprint developed a vaccine intervention model for participants to use to improve vaccination in their communities (see Figure 1).

Select the Target Population: In the early stages of COVID-19, data analysis drew attention to the massive disparities in vaccination and health outcomes that mirrored the data for other health conditions. Segmenting data allows health care professionals to hone in on specific groups of individuals and design an approach that is customized to a specific population [21].

Identify Trusted Messengers: One of the most widely cited barriers to vaccination is mistrust, both in the government and in the safety of vaccines [14]. An effective strategy to overcome this barrier is to identify trusted messengers in communities who can advocate for vaccination, provide fact-based responses to community concerns, and re-direct individuals to care if needed. These individuals may be from a variety of organizations, including faith-based organizations, schools, unions, or political groups. They might work in barber shops, beauty shops, the local grocery store, or the local health system as community health workers. 

Hone the Message: Carefully constructed messages and information about vaccination available through government-sponsored websites will not be enough to convince many individuals. Trusted messengers can focus the message on the key points of concern for a specific audience [22]. Tools such as motivational interviewing can help them explore the ideas behind the resistance to vaccination and build a greater willingness for vaccination by tailoring the message. Finally, the trusted messenger must be prepared to respond to questions related to local concerns or misinformation. 

Connect the Message and the Messenger: Difficult conversations take time, and a setting that puts individuals at ease. The 20-min physician visit is not always the ideal environment for this conversation. Fortunately, there are opportunities for this conversation in more conducive settings, such as in a church sermon, at a food bank, a high school football game, an employer site, or during a visit to the barber shop. Trusted messengers can amplify the message that patients may hear at the primary care office, thereby increasing trust in vaccines or increasing the likelihood that patients may be willing to receive a vaccine elsewhere.

Make it Easy: Making it easy to get vaccinated involves minimizing the effort and resources (specifically time or money) needed to get vaccinated. This was particularly challenging for primary care practices that may not have had COVID-19 vaccines available to patients. It is not useful for a trusted messenger to have a conversation about vaccination and then ask the individual to receive the shot at another time or place. The best practice is to combine the conversation with the vaccination, bringing vaccines to where the target population spends time. 

Nurture Relationships: Equitable health outcomes require ongoing coordination and collaboration between the community and health care. Thus, community relationships leveraged in this model must be nurtured and maintained in order to foster an ongoing trusting relationship with health systems and communities.

#### 2.1.2. Sprint Curriculum

The educational experience during the SAVE Sprint consisted of a web-based call once per week with five core agenda items: (1) the call began with a poem or quote to help participants make the transition from work into the Sprint ‘mindset’; (2) a review of participant progress to emphasize the importance of data review as a regular weekly practice; (3) a featured content expert who would share concrete tools and ideas for participants to test within their organizations (see Table 1 for SAVE Sprint curriculum topics); (4) a breakout session for deeper peer discussion and learning; and (5) each call would end with specific recommended actions for the week, as well as a short survey soliciting ideas for how to improve for the next call. The program team also hosted virtual drop-in sessions throughout the SAVE Sprint to assist with data-related questions and problem-solving.

### 2.2. Recruitment, Setting, and Participants

To recruit participants for the SAVE Sprint, we used word of mouth and partnerships with the National Association of Community Health Centers (NACHC) and the Resilient American Communities (RAC) Initiative to encourage participation. NACHC, the RAC, and the [deidentified for peer review] promoted the opportunity to join the SAVE Sprint on web-based calls through emails and in newsletters. The SAVE Sprint program team also promoted the opportunity in one-on-one calls with key leaders in different regions in the U.S.

### 2.3. Intervention Evaluation Methods

To evaluate the 10-week program, we used multiple evaluation methods to gather data throughout, and at the end of the program. During the SAVE Sprint, formative evaluation included teams submitting progress reports with data that reflected progress toward a specific aim prior to each session. Data were ideally stratified by race, ethnicity, and language and presented via run charts for the SAVE Sprint program team to review and for sharing with other teams. There were also brief post-session surveys administered at the end of the weekly calls.

Our summative evaluation method included in-depth semi-structured qualitative interviews with those teams who indicated that they were willing to share more about their SAVE Sprint experience. The interview questions focused on motivations for joining the SAVE Sprint, accomplishments attributed to the SAVE Sprint, the learning experience and content within the program sessions, suggestions for program improvements, as well as lessons learned. Participants provided verbal consent prior to being interviewed using the Zoom software platform. All interviews were recorded and transcribed. Participants received a $75 gift card as compensation for their participation in the evaluative interviews. Interviews were conducted until data saturation, or information redundancy, occurred. De-identified transcripts were thematically indexed and coded by two of the authors (ES, JS). The coding scheme included deductive codes from the interview guide, as well as inductive codes that emerged during the analysis. Two of the authors (ES, JS) independently coded each transcript and then met to review emergent themes, identify relationships between recurrent themes and concepts, and finalize the results and analysis.

The Institutional Review Board at [deidentified for peer review] declined to review this study, as it was classified as a program evaluation and quality improvement effort.

## 3. Results

**Figure 2:**
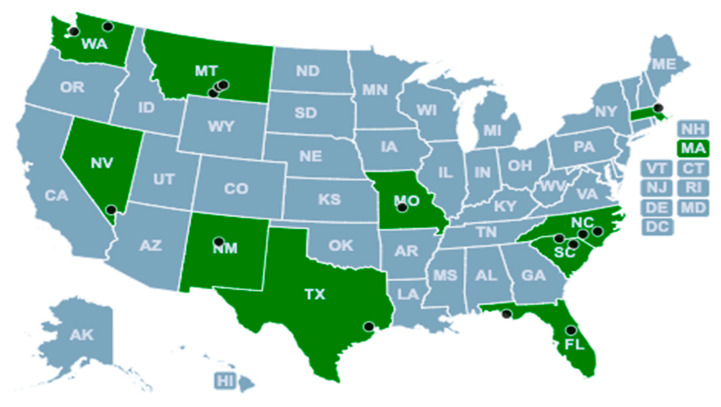
Map of Sprint Location Sites. Note: Montana (MT) had 5 sites located in the same region; 2 site markers in MT represent 2 sites instead of one.

Initially, 35 teams expressed interest in the SAVE Sprint. The program team held individual orientation calls with each team as a way to manage expectations regarding time commitment and program structure. The program team encouraged potential participant teams to include a diverse group of stakeholders, such as community health workers, physicians, staff, and patient or family partners. While two teams withdrew after the orientation calls, 13 additional teams either (1) withdrew later in the program or (2) joined calls but did not actively participate or submit assignments. Ultimately, the SAVE Sprint had a core group of 18 active participating teams from across the United States (see Figure 2), and the majority of participating organizations were community health centers. 

Most Sprint teams included representatives from the primary care team and a member of the data analysis and operations teams. Many teams also included community liaisons, such as community health workers. The team size at each site ranged from 3–8 team members. Team member presence on weekly calls varied due to the pressures of the COVID-19 pandemic; however, each of the active participating teams had 1–2 team members join weekly calls. Each team had the ability to identify the target population to increase vaccination rates over the 10-week period. The target populations varied widely, as described in Table 2. 

### SAVE Sprint Participant Evaluation

Two of the authors (ES, JS) interviewed nine team leaders who volunteered to discuss their organization’s experience with the program. Interviews lasted approximately 30 min and were conducted in March 2022, approximately three months after the program ended. As noted above, the primary aim of the SAVE Sprint was to increase COVID-19 vaccination rates for at-risk populations using a new model of change. Each site aimed to improve the vaccination rate within its chosen population of focus; the demographics of the target population, specificity of the overall aim, and target vaccination rate varied by site. 

Most of the sites reported success in meeting their goal, or progress toward their goal. For example, one community health center from the Western region was able to increase the rate of fully vaccinated Alaska Native and American Indian patients from 35% to 47%. Another community-based organization made progress increasing the vaccination rate among African American patients from 39% to 44%. For some sites, the Sprint created a sense of urgency to increase the organization’s ability to use their internal data about a specific population to drive change. For these participants, demonstrating quantitative improvement over 10 weeks was difficult, but the Sprint provided the support to understand data coupled with evidence-based strategies so that success was achieved beyond the Sprint’s 10-week timeframe. 

Sites aspiring to qualitative goals beyond vaccination reported acquisition, or building of new skills due to the SAVE Sprint, such as strengthening relationships with community partners and identifying strategies for targeting specific populations. Organizations that did not meet their established vaccination goals were still able to appreciate the educational experience and new skills learned during the SAVE Sprint. Respondents stated that one of the valuable lessons learned included taking the time to understand why people might be saying “no” to vaccination, which may look different depending on the targeted population. 

Unintended accomplishments attributed to SAVE Sprint participation were reported by all respondents. Most clinics reported that the program had beneficial effects on how staff interacted with each other, citing more professional and efficient collaboration in managing projects such as the SAVE Sprint. Respondents also noted better collaboration with external community stakeholders in terms of executing larger-scale projects. However, one interviewee noted specifically (while others alluded to the idea) that a time of crisis is not a good time to start community or partnership-building efforts. A few interviewees mentioned an increased appreciation for specific and tailored health communication and outreach initiatives. 

Interviewees suggested a few improvements for future SAVE Sprints, including a self-paced program or recorded sessions for distribution to other team members, since the program took place at a busy time of year. Another predominant suggestion was the concept of inviting more staff to SAVE Sprint conversations when available, which connected to the aforementioned self-paced aspect for future SAVE Sprints. Relatedly, some clinics also suggested inviting external stakeholders to the table, and better developing their program team prior to the start. Finally, the majority of respondents wanted a venue for maintaining the relationships they established with each other after the SAVE Sprint concluded.

## 4. Discussion

The SAVE Sprint program created a model to support community-based primary care teams in improving vaccination rates in focused populations during a pandemic. Participating teams were able to learn from each other and make progress on their project aims during a crisis, with the right support in place. The rapid engagement of participants using improvement methodology produced results, and the motivation to participate and continue was remarkable during a time of profound disengagement and burnout in health care [23]. Widespread use of improvement methodologies in everyday practice needs to become more systemically embedded as the health system recovers post-COVID and responds to pressures to reduce costs and increase quality [24,25,26].

The need to rapidly vaccinate both difficult to reach and difficult to convince U.S. populations during the COVID-19 pandemic provided incentives for health care and community-based organizations to explore more non-traditional approaches for both patient education and vaccination. Combining multiple tools into a single fast-paced initiative (the Sprint) produced a community of change agents spread across the U.S. Our findings reflect the need to develop a dependable approach to rapid-paced change methodology within the current medical culture, given the likelihood of future pandemics and disease outbreaks. 

The work of building deep and trusting relationships with communities was a theme that ran through the entire SAVE Sprint series. Vaccination proved a testing ground for the level of trust between select groups in health care and the communities they serve. While the SAVE Sprint was able to provide participants with the necessary tools and support to achieve rapid cycle change, building long-term trust is work that health care will need to continue, especially as the U.S. moves out of a public health emergency. Participants noted that relationship building is best done when not in crisis mode. Communities need to be re-engaged with local health care professionals in trusting partnerships to counteract the effects of alternate sources of information about medical issues.

## 5. Conclusions

The SAVE Sprint demonstrated that a group of community-based health care allies was able to make significant progress in a short time, not just on vaccination rates, but in building long-term trusting relationships within their communities. The SAVE Sprint model, which is an adaptation of the IHI Breakthrough Series, using a faster pace and grounded in lean principles, was judged by participants to provide a solid framework for making progress on vaccination goals and exceeded their expectations about what they could accomplish during a crisis. 

## Figures and Tables

**Table 1:** SAVE Sprint curriculum topics.

Session	Topic
1	Introduction and Kick-off: Hitting the Ground Running
2	Using Improvement and Data to Drive Culture Change
3	Building the Foundation: From Understanding History to Building Trust and Relationships—Tools for Success
4	Widening the Circle of Impact: Panel Discussion—Fireside chat
5	Trust Mapping and Community Outreach
6	Engaging the Front Line: Successful Approaches to Staff Vaccination
7	Motivational Interviewing: Techniques to Roll with Resistance
8	COVID vs Flu—Motivational Interviewing Principles Applied
9	Navigating Local Politics: Strategies for Synergy and Alignment
10	Harvest Learnings, Celebration, and the Road Ahead

**Table 2:** Participant Table.

Organization	U.S. Region	Organization Type	Target Population
1	Pacific	CBO	Russian & Ukrainian-speaking 65+
2	South Atlantic	FQHC	Males within county
3	West North Central	FQHC	Employees
4	South Atlantic	CBO	Black men
5	South Atlantic	Behavioral Health left	African American women in major metropolitan area
6	Pacific	FQHC	County residents
7	West South Central	FQHC	Youth (12–18 years old)
8	South Atlantic	Family Practice	Marginalized Populations
9	Mountain	Department of Health	Hispanic/Latinx (12–19 years old)
10	New England	Health system	Hispanic/Black (12–19 years old)
11	South Atlantic	CBO	Youth (12–18 years old)
12	Mountain	FQHC	Adults over age 55
13	Mountain	FQHC	Homeless men
14	Mountain	FQHC	Native American/Alaskan Native
15	Mountain	FQHC	Youth (12–15)
16	Mountain	FQHC	Adults over age 45
17	South Atlantic	CBO	Marginalized population
18	Mountain	CCBHC	Veterans, uninsured, crisis

Key: CCBHC—Community Clinic/Behavioral Health Center; CBO—Community Based Organization; FQHC—Federally Qualified Health Center.

## Data Availability

The data that support the findings of this study are available from the corresponding author upon request.
